# Towards a sustainable rare disease and orphan drug ecosystem in Saudi Arabia: policy insights from a multi-stakeholder workshop

**DOI:** 10.3389/fphar.2025.1583477

**Published:** 2025-05-01

**Authors:** Ghada Mohammed Abozaid, Hussain Abdulrahman Al-Omar, Abdulaziz Alrabiah, Asma AlMuaither, Amy Jayne McKnight

**Affiliations:** ^1^ Centre for Public Health, Institute of Clinical Sciences B, Royal Victoria Hospital, Queen’s University Belfast School of Medicine, Dentistry and Biomedical Sciences, Belfast, United Kingdom; ^2^ Pharmacy Practice Department, College of Pharmacy, Princess Nourah Bint Abdulrahman University, Riyadh, Saudi Arabia; ^3^ Department of Clinical Pharmacy, College of Pharmacy, King Saud University, Riyadh, Saudi Arabia; ^4^ General Directorate of National Health Policies and Economics, Saudi Health Council, Riyadh, Saudi Arabia; ^5^ C enter for Health Technology Assessment, Ministry of Health, Riyadh, Saudi Arabia

**Keywords:** orphan drugs, access, stakeholder, Saudi Arabia, challenges, rare diseases, policy

## Abstract

**Introduction:**

Rare diseases (RDs) present significant challenges worldwide, including Saudi Arabia (SA), where access to orphan drugs (ODs) is suboptimal. This study summarizes key insights from a multi-stakeholder workshop conducted in SA to explore and address challenges related to RD and OD accessibility. Strategies to improve the healthcare landscape for RDs in SA have also been recommended.

**Methods:**

A 1-day workshop, conducted at the Saudi Health Council in Riyadh, SA on 5 June 2023, gathered stakeholders from the government, private sector, pharmaceutical industry, legislators, regulators, providers, payers, academia, and insurance companies. Through a series of presentations, educational sessions, and plenary discussions, participants examined the current landscape of RDs in SA, identified barriers to accessing ODs, and recommended strategies and initiatives to improve the accessibility, innovation, and sustainability of ODs.

**Results:**

The workshop highlighted key challenges recognized by a diverse group of 59 participants, including the absence of a national strategy, absent of local RDs and ODs definitions in the Saudi context, limited awareness and understanding of RDs among healthcare professionals, delayed diagnoses, scarcity of treatment and diagnosis centers for RDs, insufficient screening and prevention programs, regulatory hurdles in approving and importing ODs, and financial constraints. These challenges significantly impact patient access to ODs, imposing additional burdens on patients, families, healthcare systems, and society.

**Discussion:**

The recommended strategies to enhance RD and OD accessibility include multifaceted approaches, such as increasing medical education and awareness, accrediting and investing in expanding the number of centers of excellence for RD diagnosis and management, streamlining regulatory processes for OD approval and importation, fostering international collaborations for knowledge exchange and capacity building, and implementing national policies to improve the affordability and reimbursement of ODs. Stakeholder collaboration is crucial to overcome the accessibility challenges of RDs and ODs. The development of comprehensive national RD strategies ensures equitable resource allocation, a national RD registry, and infrastructure improvements. These measures are vital for ensuring equitable access to ODs and the efficient provision of healthcare services in SA.

## 1 Introduction

Rare diseases (RDs), which only affect a small proportion of the population, have a significant impact on public health through increased morbidity and mortality, as well as on healthcare systems and society. The increasing global awareness of RDs has made it a priority to address their unique needs (Li et al., 2020), necessitating strategic collaboration and partnerships in research, policy, and resource allocation. More than 450 million individuals struggle with RDs (Repetto et al., 2020; Abozaid et al., 2025) and over 7,000 individual RDs have been identified (Haendel et al., 2020). Despite their low prevalence, RDs collectively present substantial challenges owing to their inherent complexity, heterogeneity, diagnostic odyssey, rarity, limited pathophysiological understanding of many conditions, and multiple treatment-related issues. These include the scarcity of effective therapies, accessibility, affordability, and availability of orphan drugs (ODs—pharmaceutical products developed specifically to treat RDs), which are currently only available for less than 5% of RDs (Kaufmann et al., 2018). This lack of treatment options leaves patients, their families, and healthcare providers grappling with the burden of managing chronic, progressive conditions.

Globally, managing RDs and ensuring OD availability remain significant challenges because many countries lack national strategies and clearly defined access policies or pathways for ODs. Although some countries have implemented policies and legislation concerning ODs, these are not universally applicable (Adachi et al., 2023). A comprehensive policy review of 194 countries and six regions (Li et al., 2020) revealed that only 92 had established OD legislations or policies (Li et al., 2020). Notable examples include the Orphan Drug Act (ODA) introduced by the United States (U.S.) Food and Drug Administration (FDA) in 1983 (Kozdrey, 2019) and the Orphan Medicine Regulation implemented by the European Union (EU) in 2000. These regulations have had a significant positive impact as evidenced by an increase in the development and approval of ODs following their enactment. For instance, the ODA has facilitated the approval of over 800 ODs, whereas only 10 were approved prior to its introduction (Pearson et al., 2022).

The lack of legislative policies and development of action plans and strategies have led to a diagnostic odyssey with limited therapeutic options that significantly impact disease management (Vashisht et al., 2023). Diagnostic odyssey, defined as the time and journey from the presumed onset of symptoms to the establishment of an accurate diagnosis, is often prolonged for RDs. Patients experience an average of 5.6 years of delays, misdiagnoses, and numerous specialist consultations before reaching a definitive diagnosis (Jefferies, 2024). The European Rare 2030 Foresight Study established goals for the diagnosis of RD ranging from 6 months to a year after the patient sought medical attention (Faye et al., 2024). Diagnostic odyssey is characterized by multiple challenges as healthcare providers may lack awareness or familiarity with RDs, leading to misdiagnoses, delayed diagnoses, multiple expensive and invasive investigations, and difficulties in accessing healthcare (Jefferies, 2024). This highlights the urgent need for extensive efforts from various stakeholders (Stoller, 2018).

Many individuals with RDs struggle to access treatment even after being diagnosed. The treatment of RDs is often hindered by regulatory and reimbursement challenges, making it less accessible and affordable (Vashisht et al., 2023). ODs face market challenges because the small number of patients does not generate sufficient revenue to cover the high costs of research, development, and production. Consequently, pharmaceutical companies are often reluctant to invest in the development of ODs, resulting in a scarcity of treatment options for many RDs (Petrova et al., 2014). To address this challenge and encourage OD investment and development, some countries offer incentives such as pricing preferences or market exclusivity, which aim to offset the initial investment. Additionally, mechanisms such as expedited review procedures and regulatory assistance are implemented to accelerate the OD designation and approval process. Despite these efforts, disparities in access to ODs persist owing to economic constraints and limitations in healthcare infrastructure (Cotlear et al., 2015).

The healthcare system in Saudi Arabia (SA) is mainly funded by the government, with multiple sectors responsible for providing healthcare services to employees and their families. Employers primarily cover those ineligibles for government-funded healthcare services with private medical insurance (Ahmed and Damrah, 2018; Al-Omar et al., 2021a). Despite the availability of substantial financial resources, the accessibility to orphan drugs (ODs) remains a challenge. These challenges include inadequate infrastructure, substandard services, insufficiently qualified healthcare professionals (Nitish, 2023), and geographical disparities in accessing healthcare through centers of excellence or referral centers between cities, rural, and remote regions. That strains the healthcare system’s capacity and delays diagnosis and treatment for RD patients (Alhawassi et al., 2018; Al-Hanawi, 2017). Another vital challenge is the ambiguity surrounding clearly defined access and registration policies (Balkhi et al., 2023). Although the Saudi Food and Drug Authority (SFDA), the licensing authority for pharmaceuticals and medical devices in SA, has published an OD designation guideline (SFDA, 2023b), it has yet to develop a country fit-for-purpose guideline for OD registration and pricing that considers the local context, including locally developed RD definitions. The ODs approval process in SA, influenced by regulatory decisions in the EU and US, are either slow or highly strict (Balkhi et al., 2023), leading to delayed access. Addressing these challenges will require not only the development of a national policy but also robust local clinical evidence and international collaboration to ensure timely access to effective therapies (Ibrahim, 2023). Despite these challenges, the health system in SA relies heavily on fostering a unified commitment toward collective action to achieve equality and equity (Al-Hanawi, 2017). Currently, the healthcare system is undergoing a massive transformation under Saudi Arabia Vision 2030, aiming to revolutionize health service delivery, improve accessibility, and enhance efficiency (Alasiri and Mohammed, 2022). Directing the healthcare system toward a preventative model of care, accountable care organizations, increased private-sector participation, and outcome-based reimbursement (Al-Omar et al., 2021b) is guided by international best practices, transformation strategic objectives, and SA local and unique context (Al-Hanawi, 2017; Alasiri and Mohammed, 2022).

The workshop aimed to foster a collaborative effort as the first multidisciplinary gathering, allowing diverse stakeholders to work together, share insights, discuss the current landscape of RDs in SA—including epidemiology, diagnostic pathways, treatment options, and existing barriers—and develop actionable strategies to enhance the accessibility of ODs and improve care for individuals living with RDs in SA. This workshop had three objectives: identifying and analyzing obstacles limiting access to ODs in SA; exploring and proposing solutions that ensure the availability and long-term sustainability of ODs; and fostering collaboration among stakeholders toward improving RD care.

## 2 Methods

A 1-day multistakeholder workshop was hosted by the Saudi Health Council (SHC) in Riyadh, SA, on 5 June 2023. The SHC is a regulatory organization dedicated to overseeing and improving the health sector in SA. It plays a crucial role in developing health policies, facilitating communication between different health organizations, and implementing strategies to promote public health and wellbeing, ensuring the highest standards of healthcare (GOV.SA, 2023).

This workshop brought together key stakeholders from various sectors, including government and private healthcare agencies, regulatory bodies responsible for drug approval and safety, the pharmaceutical industry, and patient advocacy groups. Participants included healthcare professionals, policymakers, authorities, and academic researchers. The SHC sent the invitations to stakeholders with significant contributions or relevant mandates in RD management and policy development 4 weeks in advance.

On the day of the workshop, the research team began with an informative presentation on RDs and ODs, highlighting their importance and outlining the workshop’s goals and objectives. Participants engaged in interactive, non-directive discussions based on their roles and expertise in the field. The discussion revolved around three main themes. “Accessibility theme” focused on evaluating whether patients have timely and affordable access to safe and effective ODs. Moreover, the “innovation theme” explored incentives and research motivations for developing medicines tailored to the needs of patients with RDs in SA. Lastly, the “sustainability and efficiency theme” examined mechanisms for purchasing ODs and ensuring their affordability.

### 2.1 Data collection instruments

Participants’ demographic data were collected during the workshop via a mobile device interface, using Vevox^®^ software (Golden, 2023). Following participants’ permission, key issues related to RDs, and ODs were discussed and recorded. Audio recordings and verbatim transcripts were captured during the meeting using audio technical recording equipment (Nottingham, 2018).

### 2.2 Data analyses

The transcripts were compared with the audio files to produce accurate written transcripts of the workshop discussions. Most of the transcripts were in English and very limited ones were in Arabic; the latter records were translated into English to ensure accuracy. The qualitative data were analyzed by two of the authors (GMA and AAL) following established procedures for thematic analysis, which included systematic coding and categorization, ensuring rigorous interpretation and reliability throughout the process (Braun and Clarke, 2006). Remaining discrepancies after comparing the initial findings were resolved by a third author (HAA). Numerical data were analyzed using descriptive statistics.

## 3 Results

The workshop lasted for 8 hours with a total of 59 participants from different professional backgrounds (Table 1). There were a total of 25 physicians and 18 pharmacists; and eight worked in non-clinical or administrative settings (51% government, 19% private, or 5% authority).

**TABLE 1:** Current professional background.

Characteristics	Participants (n)	%
Gender		
Male	37	63
Female	12	37
Age		
25–34	8	14
35–44	19	32
45–54	15	25
55	7	12
Current professional background		
Physician	23	39
Academic professor	12	20
Chair/member of pharmacy and therapeutic committees (PTCs)	11	19
Clinical Pharmacist	10	17
Clinical guideline expert	8	14
Health authority official	6	10
Policy maker	5	8
Key opinion leader (in a specific therapy area)	5	8
Researcher/member of research agencies	5	8
Regulator	4	7
Payer	2	3
Medical insurance	1	2
Other	13	22

Furthermore, different participants showed varied levels of understanding regarding the accessibility of ODs in SA: 11 responded being “completely green” (inexperienced or trained), 27 stated that they had “some basic knowledge,” nine indicated a “solid background,” and three identified themselves as “experts”; nine participants were missing during the session.

### 3.1 RDs and ODs accessibility challenges

During the workshop, participants identified various challenges that were categorized under six themes: policy landscape, disease burden, diagnostic odyssey, funding allocation, ODs management, and research and development (R&D) and local clinical trials.

#### 3.1.1 Policy landscape

The participants shared insights into the policy landscape challenges surrounding RDs and ODs in SA. They emphasized the complexities of the regulatory environment, particularly the SFDA’s pricing mechanisms and approval processes. One participant highlighted the SFDA’s pricing approach of considering comparative effectiveness—which is not mandated by the SFDA—rather than relying solely on external reference pricing; this necessitates the development of pharmacoeconomic guidelines that are adapted to the local Saudi context. Another participant pointed out that the 2-year re-evaluation requirement for drug pricing adds further complexity to reimbursement procedures, signaling the need for more streamlined policies and alignment with official mandates.

A participant also noted that while the draft guidelines incorporated external pricing factors, there was insufficient local data and limited understanding of RD and OD complexities among healthcare providers and policymakers, which hindered this strategy and subsequently impacted the regulatory efficiency and patient access to appropriate care. This lack of clinical data and understanding deficits often lead to misdiagnoses and inadequate treatment plans.

Furthermore, physicians face additional regulatory and procedural barriers, including the SFDA’s lengthy approval processes and complex administrative requirements that delay OD registration and importation. Pharmaceutical companies echoed these concerns, explaining that local regulatory requirements are sometimes misaligned with international standards, which subsequently delays approvals and obstructs the timely availability of ODs in SA. The exclusion of Saudi patients from global clinical trials further exacerbates these issues, affecting the representativeness of Saudi population data and restricting their access to potentially life-saving therapies.

The lack of strong patient representation in policymaking further aggravates these challenges, as mentioned by a participant, making it difficult for individuals and families to advocate for the necessary reforms. Underfunding or developmental constraints may limit the impact of advocacy groups and patient organizations in SA, despite their potential to influence policy and raise awareness.

#### 3.1.2 Disease burden

Regarding disease burden management, participants emphasized the need for well-established patient registries, comprehensive coding systems, and consistent classification mechanisms for RDs. They noted that without registry data or with insufficient registry data, tracking patients, estimating RD prevalence, or allocating sufficient resources for patient support is difficult. Additionally, registries are crucial for the screening and prevention process, which helps pharmaceutical companies target populations for clinical trials and research. The Saudi Pediatric Neurology Society (SPNS) representative emphasized the need for further initiatives to support screening and prevention programs.

While determining the prevalence and incidence of RDs remains challenging, some participants highlighted the availability of registry data for specific conditions, such as the National Newborn Screening Program, which identifies certain metabolic and genetic disorders, such as phenylketonuria (PKU), congenital hypothyroidism, and sickle cell disease. This program screens approximately 16–17 inborn metabolic diseases, that affects approximately one in every 500–1,000 newborns. However, funding limitations have stalled progress in certain programs, as mentioned by one participant who had been working on the “Birth Deficit Registry” since 2015. Another participant recommended initiating a registry for lower-cost diseases such as Multiple Endocrine Neoplasia (MEN 2), where preventive measures such as thyroidectomy could yield significant long-term savings.

However, the absence of specific RD codes in classification systems [such as the International Classification of Diseases, 11th Revision (ICD-11)] in SA poses significant challenges in terms of misclassification, underreporting, and accurate tracking. Participants emphasized that such coding gaps, combined with a lack of consensus on the definitions of RD and insufficient awareness, limit the ability to effectively manage healthcare resources; it can also lead to fragmented patient care due to inconsistencies in the medical records, a lack of interoperability between healthcare systems, and variations in recordkeeping standards.

As participants noted, these limitations not only hinder policymakers who rely on accurate data for strategy development and funding allocation but also deter industry investment in RDs because of uncertainties about the market size and return on investment. Given that SA serves as a key pricing reference for the Gulf Cooperation Council (GCC) countries, pharmaceutical companies wish to prioritize registering ODs in SA.

#### 3.1.3 Diagnostic odyssey

Participants highlighted the extensive challenges encountered by patients with RD and their families in obtaining accurate diagnoses, a journey often described as a diagnostic odyssey. Owing to the nonspecific and often ambiguous symptoms of many RDs that can mimic more common conditions, patients often endure numerous visits to different specialists and undergo multiple tests before receiving a definitive diagnosis. This prolonged journey delays treatment and exacerbates patients’ feelings of isolation and anxiety.

One physician stated that a key issue was the misdiagnosis or underdiagnosis of RDs. They recounted a case in which a child was misdiagnosed with type 1 diabetes mellitus, when in reality, the child was suffering from Wolfram syndrome, a very rare disorder. Therefore, increasing awareness of RDs, particularly those affecting children, will be a vital step toward improving diagnostic accuracy. An academic from a Saudi University stressed the role of public awareness, healthcare provider training, genetic counselling, and social media in promoting early detection and directing patients to the appropriate facilities for timely diagnosis.

Numerous participants emphasized the limited awareness and knowledge of RDs among healthcare providers, patients, and families, which significantly contributes to diagnostic challenges due to the infrequency of these diseases. This lack of knowledge frequently leads to delayed referrals, improper management, and significant delays in accessing specialized care. Indeed, as mentioned by participants, families and patients lack the necessary guidance, support, or resources to effectively navigate the intricate diagnostic and treatment processes. One physician stated that despite the efforts to educate, some patients and families do not fully appreciate the available services. For instance, parents may discontinue clinic visits after realizing that frequent visits are required for treatment. They emphasized the importance of actively engaging with patients to build awareness and understanding. To address these gaps, the participants recommended integrating RDs and ODs education into medical college curricula to equip healthcare providers with the essential diagnostic skills. Additionally, participants suggested implementing public awareness campaigns to educate families and patients about RDs.

Recognizing these challenges, the Saudi Commission for Health Specialties has taken significant steps to enhance healthcare providers’ competencies. Through the implementation of specialized training programs, the Commission has strengthened the recognition and management of RDs, equipping healthcare professionals with the necessary expertise to improve diagnostic accuracy and patient outcomes.

The International Classification of Diseases (ICD) system’s lack of specific RD codes poses a significant barrier to early diagnosis and research efforts. One participant suggested that adopting other countries’ coding systems and aligning them with the national RD definitions could enhance early diagnosis, prevention programs, and research. Furthermore, screening and prevention programs are essential for improving the accessibility of ODs. However, these initiatives face significant challenges, including a need for dedicated infrastructure, funding, and a comprehensive approach for gathering data. A public health authority representative remarked, “We are struggling to gather the necessary data to develop effective screening programs for RDs, which is crucial to preventing these diseases and is a major future economic goal.” A physician recommended starting in areas with a higher prevalence of actionable diseases, considering the local relevance and policies.

Accordingly, the National Strategic Committee for Genetic Diseases oversees three subcommittees: the Newborn Screening Committee, Prenatal Screening Committee, and Premarital Screening Committee. The Premarital Screening Program mandates the identification of carriers of conditions, such as sickle cell anemia and thalassemia while providing genetic counselling to prospective couples. However, prenatal screening, while beneficial, encounters geographic, economic, and awareness barriers in rural areas. Participants noted that these programs can significantly shorten the diagnostic odyssey by promoting early diagnosis and awareness, although their success relies heavily on proactive outreach and data sharing.

Another obstacle is the limited number of centers of excellence, equipped with advanced diagnostic tools, genetic testing capabilities, and specialized resources, for RD diagnostics and care. The absence of such centers hampers physicians’ ability to diagnose accurately. Participants noted that geographic distribution compounds this issue, as patients in rural or remote areas experience more significant access challenges than those near urban centers. Patients in these areas often must travel long distances to receive appropriate diagnoses and treatment.

One participant highlighted that infrastructure inequities and mismatch between diagnosed patients and clinic availability negatively affect the quality and continuity of RD care nationwide. This situation frequently leads to treatment non-adherence or cessation, especially for patients facing logistical and financial challenges. Additionally, some participants noted that RD diagnoses could cause social stigma, leading to social isolation and discrimination, which hinder the development of a well-informed patient community.

#### 3.1.4 Funding allocations

Participants highlighted significant challenges in funding allocations for RDs and ODs in SA, emphasizing the need for a comprehensive national strategy to ensure consistency in the availability, affordability, and accessibility of treatments.

Health system funding is often directed toward more common diseases, leading to RDs and ODs being underfunded and overlooked. This funding gap affects critical areas, such as high-cost therapies, lifelong treatments, supportive care, and essential yet costly services such as genetic testing, which are vital for early detection, but remain out of reach for many families. Indeed, some families face significant financial struggles, as one physician noted, and many parents cannot afford regular clinical visits. This financial strain negatively affects treatment adherence and the overall outcomes.

The funding gap is further compounded by insufficient registry data, limited clinical evidence, shorter study durations, reliance on surrogate endpoints, and a lack of randomized controlled trials, all of which collectively degrade the quality of RD research and clinical trials. Consequently, as one participant noted, the financial burden often falls on patients because of insufficient funding for R&D programs, which affects both their scope and long-term sustainability. Furthermore, for pharmaceutical companies, significant investments in R&D for ODs further increase costs, making these drugs prohibitively expensive for both patients and healthcare systems. Addressing these challenges requires strategic reallocation of resources and enhanced research frameworks to ensure better outcomes for patients with RD.

Participants emphasized that increasing public awareness is key toward closing these gaps. A well-informed community can advocate for dedicated budgets, improved funding, and better resource allocation for RDs. By fostering awareness and adopting a unified strategy, prioritizing RDs and improving the lives of the affected patients and their families will be possible.

Participants further noted the minimal involvement of insurance companies, with the government currently bearing most of the funding responsibilities. However, insurance companies are expected to play a larger role as Saudi Arabia’s healthcare system evolves. An insurance representative highlighted the need for creative approaches and enhanced financial resources, recommending collaboration between the public and private sectors to explore models such as Managed Entry Agreements (MEAs) to handle uncertainties and/or financial risks. Another participant opined that such a collaboration could make ODs more accessible without compromising their financial viability. Participants also recommended investigating value-based healthcare models that align funding with patient outcomes, thereby potentially enhancing the management of RDs and ODs.

#### 3.1.5 Complexities of managing ODs

Since 2017, the SFDA has made significant advancements in the regulatory oversight, approval processes, and market access of ODs by forming an expert team dedicated to ensuring their quality, safety, and efficacy. This effort led to the launch of specific guidelines for OD designation in 2023 (SFDA, 2023b). The guidelines introduced multiple access pathways such as breakthroughs designations, bridging programs, verification processes, and accelerated approvals. SFDA representatives noted significant improvements in ODs market access, citing reduced registration times as short as 8 months - a significant improvement from the previous span of 4–5 years - through initiatives such as the “early dialogue” approach (Thanimalai et al., 2021). Additionally, hospitals can also procure unregistered drugs directly, although the SFDA disclaims liability for adverse effects in these cases.

The SFDA representatives highlighted ongoing initiatives aimed at improving access to high-cost therapies, such as using real-world data to assess the pharmacoeconomic value of high-tech drugs. This approach involves adjusting drug prices after two and 5 years based on hospital data to ensure their continued value. They also proposed a value-based patient access model to enhance access to treatment; however, this initiative faced resistance from pharmaceutical companies. The center for health technology assessment (CHTA) emphasized its critical role in reimbursement decisions, recommending participation in MEAs to mitigate the financial risks associated with costly therapies that have uncertain outcomes. In addition to these challenges, there is often a lack of clear guidelines and criteria for reimbursement, leaving many patients unable to access existing treatments. One participant described this situation as a “tragedy,” where patients are aware of the availability of lifesaving therapies but cannot afford them due to funding constraints. These issues highlight the urgent need for systemic changes to address the gaps in access and affordability.

A participant described the management of ODs as largely “*ad hoc*,” stressing on the need for a more structured national forecasting system to better anticipate distribution needs and ensure consistent availability. Another participant mentioned that advanced technologies could strengthen logistics for high-tech drugs that face significant supply chain challenges. Another participant highlighted that limited information about ODs, including their availability and accessibility, leaves patients and families with little awareness and understanding of available treatment options. This can lead to underutilization of existing medications.

The National Unified Procurement Company (NUPCO) is responsible for managing the procurement, logistics, and supply chain for pharmaceuticals, medical devices, and governmental hospitals. NUPCO was established in 2009 and owned the Public Investment Fund with the goal of achieving cost efficiency. However, it faces coordination challenges across healthcare sectors, especially for non-formulated or unregistered ODs, which delays availability. A NUPCO representative called for stronger collaboration with pharmaceutical companies to predict sector-wide drug demands and reduce delays, thereby improving access for patients and families.

Another participant mentioned that a comprehensive ODs management framework would require alignment among stakeholders, including the SFDA, NUPCO, CHTA, and healthcare providers. Participants collectively underscored the need for better coordination, forecasting, and technology adoption to enhance OD availability and ensure that the management process aligns with the needs of patients and the evolving healthcare landscape in SA. This can be achieved by generating local evidence through either real-world data or clinical trials in the SA population and streamlining operations for optimal OD management.

#### 3.1.6 Critical challenges in R&D for RDs and ODs

The lack of specialized infrastructure and trained researchers exacerbates these challenges in R&D. SA has very few advanced laboratories and research facilities as well as trained researchers with expertise in RDs to address the specific demands of OD development. One participant remarked, “Most of us are clinicians who have no background in research, and those who are experts as researchers are few; they really cannot cover all RDs.”

One participant highlighted the need for a national strategy aligning R&D with real-world data to attract global companies and foster innovation in RD therapies. Another participant opined that the local co-development and manufacturing of therapies, supported by current patents, could enable early-stage clinical trials for RDs. Another suggested an initiative involving the SFDA and local industries to foster OD R&D, expressing optimism that recent agreements could propel local drug development.

Recruiting for clinical trials presents unique challenges, particularly because of the genetic diversity of the Saudi population, which introduces variability in disease presentation and treatment responses. This diversity often necessitates larger sample sizes for statistically significant results. However, the absence of comprehensive registry data hinders the identification of eligible participants and limits our understanding of disease epidemiology. Furthermore, although establishing a registry would be beneficial, administrative burden and privacy concerns pose significant obstacles to the creation of such a registry.

Participants also highlighted the ethical considerations when conducting clinical trials, particularly in vulnerable populations with RD. The lack of patient advocacy and limited awareness of RDs further reduce the momentum for research initiatives and funding, slowing the introduction of new therapies and limiting patient access to information on their conditions and potential treatment options. One participant noted that social media and public health campaigns as key tools for boosting public awareness and encouraging early intervention in RDs research.

### 3.2 Priorities for action to optimize RD patient care in Saudi Arabia

The multi-stakeholder workshop highlighted the interdependencies and intertwined challenges faced by stakeholders, and participants from various sectors established critical priorities to optimize care for patients with RD and access to ODs in SA.

**FIGURE 1:**
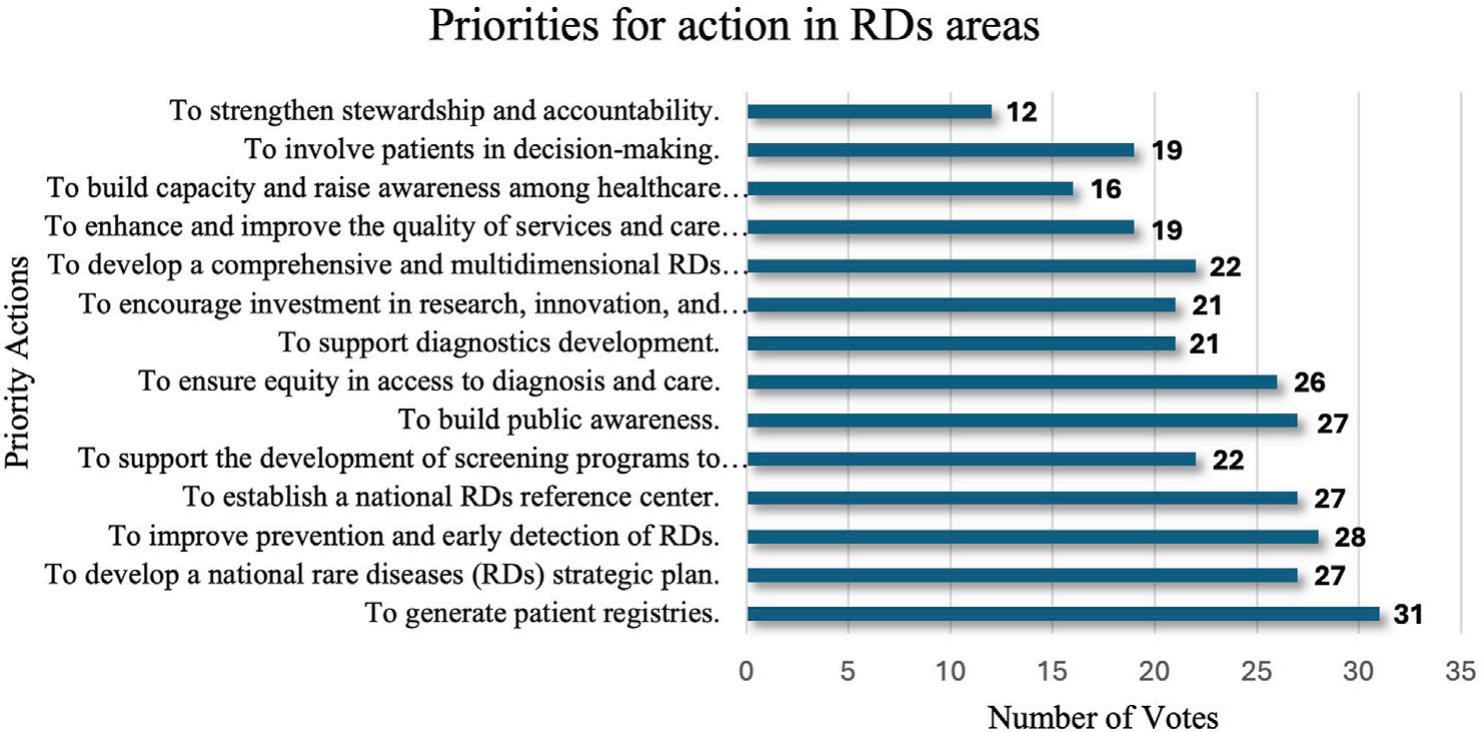
Priorities for action in RD areas.

Through a structured voting process, stakeholders highlighted 14 essential actions for RDs’ areas that require immediate attention (Figure 1).

The top priority, as indicated by the votes, was the establishment of comprehensive patient registries deemed vital for understanding disease prevalence, guiding research, and supporting healthcare planning. There was also an emphasis on developing a national RD strategic plan, regarded as fundamental to organizing and directing long-term efforts to tackle the unique challenges of RD care in a structured manner.

Early detection and prevention measures were also highly prioritized, emphasizing the need to improve diagnostic timelines and patient outcomes. Only then did the participants focus on establishing a national RD reference center, which was envisioned as a central hub for RD resources, guidance, and patient support. Additional priorities included strengthening stewardship, accountability, and patient involvement in the decision-making processes. Stakeholders also emphasized capacity building among healthcare workers, fostering public awareness, and ensuring equity in diagnostic access and care quality. Collectively, these priorities underscore a unified approach that leverages the data infrastructure, strategic planning, early intervention, and broad-based engagement to foster a sustainable and equitable RD healthcare ecosystem in SA.

## 4 Discussion

The RD and OD ecosystem (Tafuri et al., 2022) presents significant healthcare challenges worldwide (Adachi et al., 2023), requiring collaboration among multiple stakeholders, robust policies, and streamlined regulatory pathways to improve patient outcomes. Like many other countries, SA faces barriers in RD management and OD accessibility, including regulatory hurdles, financial constraints, diagnostic delays, and the absence of a national strategy. To author’s knowledge this is one of first studies in Saudi Arabia to examine how healthcare providers, policymakers, pharmaceutical companies, researchers, and patients work together to address these challenges while identifying key obstacles and proposing policy recommendations to enhance OD availability and accessibility.

### 4.1 Regulatory and policy barriers in OD accessibility

The absence of a structured national strategy for ODs in SA contributes to fragmented policy implementation, delays in drug approvals, and inconsistencies in reimbursement policies. Workshop participants emphasized the Saudi Food and Drug Authority (SFDA)’s complex regulatory processes, particularly the lengthy drug approval timelines, pricing restrictions, and re-evaluation requirements every 2 years, which create uncertainty for pharmaceutical companies and limit patient access to essential treatments. These challenges mirror global trends (Gilabert-Perramon et al., 2017), as observed in Canada and Australia, where limited pharmacoeconomic data, complicated approval processes, and rigid pricing frameworks delay OD availability (Tafuri et al., 2022; Adachi et al., 2023; Szanowni, 2024; Gentilini et al., 2025).

Comparatively, the Orphan Drug Act (ODA) in the United States (US) and the Orphan Medicinal Products Regulation in the European Union (EU) have significantly accelerated OD development through financial incentives, expedited regulatory pathways, and extended market exclusivity (Saini et al., 2024). The EU’s initiatives encompass the creation of the Orphanet platform and the use of ORPHA codes (Aymé et al., 2015), which are used in standardizing RD classification, enhancing research coordination, and improving patient access. The Breakthrough Therapy Designation (BTD) in the US and the PRIME program in the EU have further streamlined approval processes, prioritizing drugs with high therapeutic potential (Gentilini et al., 2025). Implementing a similar accelerated framework in SA—tailored to local needs—could significantly enhance OD accessibility, incentivize pharmaceutical investments, and reduce approval delays.

A key issue raised during the workshop was the misalignment of local regulatory policies with international standards. Establishing expedited pathways for high-priority therapies, fast-track access committees (Rezaie and Singer, 2010), and aligning local regulations with international standards could help reduce delays and improve accessibility while maintaining financial sustainability. While SFDA considers external reference pricing, it lacks robust pharmacoeconomic guidelines adapted to SA’s disease burden. This misalignment creates delays in drug pricing negotiations and reimbursement approvals. Incorporating health technology assessments by the CHTA into ODs access pathways for evaluating cost-effectiveness and clinical value is highly necessary.

Lessons from the EU and Canada suggest that integrating Multi-Criteria Decision Analysis (MCDA) into regulatory processes could improve transparency and facilitate evidence-based decision-making (Gongora-Salazar et al., 2023). Moreover, enhanced collaboration among regulatory bodies, clinical experts, and pharmaceutical companies, combined with streamlined SFDA registration processes and CHTA reimbursement support, can significantly improve drug access. Early dialogues (Thanimalai et al., 2021) and generation of local evidence are essential for reducing risks, accelerating drug availability, and driving innovation (Kontoghiorghe et al., 2014).

### 4.2 Challenges in RD diagnosis and disease burden management

Timely and accurate RD diagnosis remains one of the most significant challenges in SA, with limited awareness among healthcare professionals, inadequate genetic screening programs, and an absence of national patient registries contributing to prolonged diagnostic odysseys.

Workshop participants emphasized that the lack of trained specialists, underutilization of advanced diagnostic tools, and geographic disparities (Griggs et al., 2009) further exacerbate delays. Infrastructure gaps, including fragmented patient records, pose significant challenges to RD management. Similar diagnostic challenges have been documented in the European Rare 2030 Foresight Study, which highlighted that delayed diagnosis remains a major barrier in RD management worldwide (Kole et al., 2021). Establishing centers of excellence (Soverini et al., 2024; Aparna and Ratnakar Kini, 2024) and diagnostic laboratories has been identified as a critical step toward centralizing expertise and improving early detection.

The lack of a proper RDs coding system in SA creates major challenges in healthcare system. Without standardized codes, healthcare providers struggle to track RDs, leading to gaps in medical records, inconsistent patient care, and difficulties in managing resources efficiently. Countries such as France and Germany have integrated specific International Classification of Diseases (ICD) codes for RDs, improving data collection, resource allocation, and patient tracking (Broich et al., 2024). Implementing a similar system in SA could enhance epidemiological surveillance, support early diagnosis, and facilitate research collaborations.

Furthermore, stakeholders emphasized the need for comprehensive national registries, which are critical for tracking disease prevalence, supporting clinical trials, and guiding treatment decisions. Countries such as Japan and the UK have successfully implemented national RD registries, improving early diagnosis and targeted treatment strategies (Broughan et al., 2024; Furusawa et al., 2019). Establishing a centralized registry in SA would significantly improve healthcare planning, enable real-world evidence (RWE) generation, and attract international research collaborations.

The Saudi Pediatric Neurology Society (SPNS) emphasized the need to enhance screening and prevention programs to address the high prevalence of genetic disorders caused by consanguinity. The high consanguinity rates in SA significantly contribute to the prevalence of genetic disorders, highlighting the need for robust screening and prevention programs (Stoller, 2018). Comprehensive screening initiatives coupled with genetic counseling, are essential for addressing this burden. Sustained funding through multiyear budgets is required to ensure the stability of these initiatives and enable the long-term evaluation of their effectiveness (Griggs et al., 2009).

Finally, policymakers must periodically revisit and update RD and OD definitions to ensure alignment with global standards and strategic priorities (Phillips, 2013). A dynamic approach to policy adaptation will support a more efficient healthcare system, fostering innovation in RD treatments and ensuring better outcomes for patients with rare diseases in SA.

### 4.3 Financial and economic constraints in OD affordability

Affordability remains a major barrier to OD access in SA, with high drug costs driven by global trade regulations (Kontoghiorghe et al., 2014), monopolistic pricing practices, and research and development (R&D) expenses. Collaborative efforts between the private sector and international research institutions can offset the high costs of R&D, advance innovative projects, and ensure equitable access to ODs. Despite advancements in local drug manufacturing, the high costs of both generic and advanced therapies (Schweitzer and Lu, 2018) continue to hinder accessibility (Vincent, 2020); for instance, Spinraza^®^ costs 3,947,62.5 SAR (SFDA, 2023a).

Currently, the government bears most of the financial burden, with minimal involvement from insurance providers. Policies that promote subsidies, insurance support, market competition, and innovative pricing models are essential for improving affordability. Workshop participants proposed alternative financing models, such as public-private partnerships, value-based pricing, and risk-sharing agreements, to improve affordability without compromising sustainability.

Several high-income countries have adopted managed entry agreements (MEAs), which allow conditional reimbursement based on real-world outcomes (Thanimalai et al., 2021). Implementing MEAs in SA could help mitigate financial risks while ensuring patient access to high-cost, innovative therapies (Al-Omar et al., 2021b). Additionally, integrating value-based pricing mechanisms (Al-Omar et al., 2021a), where drug costs are linked to patient outcomes, could create a more sustainable pricing model for ODs.

Another major financial barrier is the lack of dedicated RDs and ODs funding allocations. Many countries have allocated budgets for RDs and ODs (Ng et al., 2024). For example, the EU supports RDs and ODS through several programs, such as Horizon Europe (European Commission, 2025a), which provides research grants, and the EU4Health Programme (European Commission, 2025b), which allocates funds for diagnostics, patient registries, and specialized treatment access. A similar dedicated RDs and ODs funding program in SA, supported by government grants, and research funding agencies, could enhance long-term financial sustainability.

### 4.4 Research, innovation, and local pharmaceutical development

The lack of local OD research and manufacturing remains a significant barrier in SA’s healthcare landscape. Participants emphasized that R&D constraints, limited clinical trials, inadequate funding, insufficient research expertise among clinicians and reliance on imported treatments contributing to higher costs and delayed market access. Addressing these challenges through public-private partnerships between pharmaceutical companies, academic institutions, and regulatory bodies could foster innovation in RDs treatments by providing the necessary financial and technical support. In this context, SA’s Vision 2030 (Alasiri and Mohammed, 2022) initiative presents a strategic opportunity to integrate RD research into the national innovation strategy, encouraging domestic drug development and reducing reliance on external suppliers, ultimately improving access to life-saving therapies.

Recruiting patients for clinical trials in SA remains a major challenge due to the country’s genetic diversity and limited patient registry infrastructure. Implementing adaptive clinical trial designs, which allow for smaller patient cohorts and real-time data adjustments, could help overcome these barriers by accelerating research timelines and improving trial efficiency. Additionally, incentivizing local pharmaceutical investments through tax benefits and regulatory waivers could encourage greater industry participation in OD development. Considering this, promoting SA’s participation in both global and local clinical trials is crucial for generating locally relevant data, reducing reliance on external evidence, and ensuring that research outcomes align with the unique needs of the Saudi population.

### 4.5 Improving RD awareness, education, and advocacy

A recurring theme throughout the workshop was the lack of awareness and educational initiatives related to RDs and ODs among healthcare professionals, patients, and policymakers. The low representation of health authority officials (10%) and policymakers (8%) among the participants reflects this issue and points to a broader challenge of regulatory engagement in specialized therapeutic areas such as RDs and ODs. Additionally, many physicians lack the necessary training to diagnose and manage RDs effectively, resulting in misdiagnoses and delayed interventions. To address this, stakeholders recommended integrating RD education into medical school curricula and continuous professional development programs.

Countries such as Canada (CORD, COfRD, 2021) and the UK (Government UK, 2021) have successfully implemented RD training modules, leading to improvements in early detection and management. Similarly, public awareness campaigns, and patient advocacy programs, could help bridge knowledge gaps, reduce stigma, promote early diagnosis, and empower RD patients to navigate the healthcare system more effectively.

### 4.6 Strategic recommendations for Saudi Arabia

Based on workshop findings several policy recommendations were proposed to enhance RD management and OD accessibility in SA:

1. Develop a National RD Strategy: Establish a dedicated policy framework to guide RD research, funding, and treatment accessibility.
2. Accelerate Regulatory Reforms: Introduce fast-track approval pathways, adaptive pricing models, and harmonized reimbursement policies aligned with international standards, while considering the Saudi local context.
3. Expand Centers of Excellence: Invest in specialized diagnostic and treatment centers to improve early detection and multidisciplinary care.
4. Enhance Data Infrastructure: Develop a national RD registry and integrated electronic medical records to support real-world data collection and clinical trial recruitment.
5. Foster Public-Private Partnerships: Encourage collaborations between government, industry, and academia to drive local OD innovation and manufacturing.

### 4.7 Limitations

Although this study offered valuable insights into the challenges and enablers of OD access and RD management in SA, it had several limitations. First, the findings were based on a single-day workshop, which may have limited the depth of discussion, unintentionally marginalized certain voices or ideas, and contributed to participant fatigue. Second, the underrepresentation of certain stakeholders, such as patient populations and nurses, may have limited the study’s findings and potentially impacted the generalizability of the findings across all RD and OD stakeholders in SA. Third, the built-in anonymity of Vevox^®^ may have discouraged participants from participating in the workshop.

## 5 Conclusion

The workshop highlighted key areas for improving the RD and OD ecosystem in SA, emphasizing targeted education, regulatory efficiency, patient representation, and alignment with international standards. Ensuring equitable access to ODs and enhancing RD management requires a comprehensive, multi-sectoral approach that includes evidence-based policies, investment in research and innovation, streamlined regulations, and stronger patient advocacy.

By implementing these recommendations and fostering collaborative efforts among stakeholders, SA can overcome existing challenges, enhance healthcare efficiency, and improve patient outcomes. Workshop attendees recognized the importance of ongoing discussions and frequent meetings to drive sustainable improvements, ultimately positioning SA as a leader in RD care while improving the quality of life for patients with rare conditions.

## Data Availability

The raw data supporting the conclusions of this article will be made available by the authors, without undue reservation.

## Abbreviations

AA, Abdulaziz Alrabiah; AAL, Asma AlMuaither; AM, Amy Jayne McKnight; CHTA, Center for health technology assessment; EU, European Union; FDA, Food and Drug Administration; GCC, Gulf Cooperation Council; GMA, Ghada Mohammed Abozaid; HAA, Hussain Abdulrahman Al-Omar; ICD-11, International Classification of Diseases, 11th Revision, MEAs, Managed Entry Agreements; MEN 2, Multiple Endocrine Neoplasia; NUPCO, National Unified Procurement Company; ODA, Orphan Drug Act; ODs, orphan drugs; PKU, Phenylketonuria; PNU, Princess Nourah Bint Abdulrahman University; RDs, Rare diseases; R&D, research and development, SA, Saudi Arabia; SFDA, Saudi Food and Drug Authority; SHC, Saudi Health Council; SPNS, Saudi Pediatric Neurology Society; U.S., United States.
